# Evaluation of an educational program for nurses on the care coordination of pleural mesothelioma patients: A pilot randomized controlled trial

**DOI:** 10.1111/jjns.70043

**Published:** 2026-01-27

**Authors:** Yasuko Nagamatsu, Wakanako Ono, Yumi Sakyo, Edward Barroga, Keiko Hosokawa, Minako Ito, Shino Matsuura, Taiki Fukujin, Yoko Maehara, Masaya Ito, Haruhiko Mitsunaga, Satomi Nakajima, Nobukazu Fujimoto, Kazunori Okabe

**Affiliations:** ^1^ St. Luke's International University Tokyo Japan; ^2^ Tokyo Metropolitan University Tokyo Japan; ^3^ Showa Medical University Tokyo Japan; ^4^ St. Luke's International Hospital Tokyo Japan; ^5^ Tokyo Medical University Tokyo Japan; ^6^ Toho University Chiba Japan; ^7^ Hyogo Medical University Hyogo Japan; ^8^ Bell Land General Hospital Osaka Japan; ^9^ National Center of Neurology and Psychiatry Tokyo Japan; ^10^ Nagoya University Nagoya Japan; ^11^ Musashino University Tokyo Japan; ^12^ Okayama Rosai Hospital Okayama Japan

**Keywords:** ANOVA, care coordination, education, mesothelioma, nurse, randomized controlled trial

## Abstract

**Objective:**

This study aimed to evaluate the effects of the *care coordination of pleural mesothelioma program* (CCOM program), an educational program that we developed for nurses to improve their knowledge, attitude, and confidence on the care coordination of pleural mesothelioma (PM) patients.

**Methods:**

In this randomized controlled study relative to the CCOM program, we measured the self‐reported total scores of knowledge, attitude, and confidence of nurses before (pre‐test) and after (post‐test) the CCOM program. The CCOM program consisted of a care guide and nine study videos featuring various aspects of PM (2 h 15 min) and a face‐to‐face workshop (3 h 30 min). Sixty participants were randomly assigned to the intervention group (*n* = 30; with program) and control group (*n* = 30; without program). Fifty‐eight participants completed the study (intervention group, 28; control group, 30). The total scores of knowledge, attitude, and confidence at pre‐test and post‐test were compared using one‐way repeated measures analysis of variance (ANOVA).

**Results:**

At pre‐test, the groups showed no significant differences in age, experience of taking care of PM patients, and working department, except for the years of experience as a nurse. The CCOM program improved the knowledge, attitude, and confidence in the intervention group, whereas the control group showed stability in the scores. Repeated measures ANOVA showed significantly greater beneficial changes in the intervention group on main measurement scores.

**Conclusions:**

The CCOM program was effective in improving the knowledge, attitude, and confidence of nurses on the care coordination of PM patients.

## 1 INTRODUCTION

Pleural mesothelioma (PM) is a rare malignancy (Beasley et al., 2021) which is almost exclusively caused by asbestos exposure (van Zandwijk et al., 2022). The survival period after diagnosis is as short as 8–19 months (Asciak et al., 2021; Bou‐Samra et al., 2023; Fournel et al., 2022; Guzmán‐Casta et al., 2021; Hasegawa et al., 2024; Kang et al., 2021; Kerrigan et al., 2022). PM causes a series of debilitating symptoms (Nagamatsu et al., 2022a) and additional distress as a victim of the asbestos industry (Guglielmucci et al., 2018). The number of annual deaths from PM in Japan is about 1600, and this number has been increasing in recent years (Japan Ministry of Health, 2025). The scarcity of specialized PM practitioners in Japan often requires patients to travel domestically within Japan to receive appropriate treatment and management services. When these patients return to their own hometown in Japan, their treatment and care are often discontinued.

A previous study of bereaved family members reported that PM patients rarely achieved a good quality of life in the terminal stage at home, which is related to the failure of care provision (Nagamatsu et al., 2022a). This indicates that receiving quality care when a patient becomes critical is crucial at home for PM patients. However, it is very challenging to provide quality care in a timely manner to PM patients because of several reasons. Firstly, PM progresses rapidly. Secondly, care provided in acute care hospitals and in home‐based settings operates independently and remains fragmented (i.e., fragmented care), which results in a lack of continuity. Care coordination is expected to improve the fragmentation of care and bring consistency, continuity, and coordination to care.

Fragmentation of care also hinders nurses in acute care hospitals from understanding the care needs of PM patients living at home after discharge, leading to failed care coordination. Finally, there is a limited number of medical institutions providing treatment and care for PM. A meta‐analysis previously reported that cancer care coordination improved 81% of the outcomes, including screening, measures of patient experiences with care, and quality of end‐of‐life care (Gorin et al., 2017). With the growing complexity of the needs of patients, nurses must play a substantial role in the care coordination of patients with complex care needs (Karam et al., 2021). However, previous reviews have reported on only some conditions for achieving successful interprofessional care coordination such as having a shared vision (Khatri et al., 2023), using guidelines (Lisy et al., 2021), frequent communication and clinical interaction (Khatri et al., 2023; Lisy et al., 2021), recognition of the skills of other professionals (Khatri et al., 2023), defining the roles of each health care provider (Lisy et al., 2021), providing referrals (Khatri et al., 2023), producing summaries (Khatri et al., 2023), and conducting trainings (Khatri et al., 2023; Lisy et al., 2021). To promote care coordination in PM care, a care guide was developed previously (Nagamatsu & Sakyo, 2023). However, to fully accelerate care coordination, it is necessary to share the care needs of patients and their families between hospitals and communities, as well as to confirm the individual roles of nurses in providing patient‐centered care.

In this study, we developed and conducted an educational program for nurses on the care coordination of PM patients (i.e., CCOM program). Our primary aim is to evaluate the effects of the CCOM program on the knowledge, attitude, and confidence of nurses on the care coordination of PM patients.

## 2 THEORETICAL FRAMEWORK

The theoretical model of self‐efficacy by Bandura and Walters (1977) and Bandura (1986), which is based on the social learning theory, was used as the framework for the present study. *Perceived self‐efficacy* is concerned with people's beliefs in their capabilities to exercise control over their own functioning and over events that affect their lives (Bandura, 1994). People's beliefs in their efficacy are developed from four main sources of influence: *mastery experiences, witnessing people similar to oneself manage task demands successfully*, *social persuasion that one has the capabilities to succeed in given activities*, and *inferences from somatic and emotional states indicative of personal strengths and vulnerabilities* (Bandura, 1994). Multiple studies have reported the effectiveness of self‐efficacy in nursing education (Arslan et al., 2018; Cayır & Ulupınar, 2021). Nurses face limited opportunities to care for PM patients with complex conditions. In the present study, we used Bandura et al.'s theoretical model of self‐efficacy to implement an educational program to improve nurses' care coordination skills. The program provides opportunities to learn from and cooperate with other participants in the care coordination of PM patients.

## 3 METHODS

### 3.1 Study design

This study was an open‐labeled randomized controlled trial (pre‐test and post‐test design) with two parallel groups at 1:1 ratio. The trial protocol was registered with the University Hospital Medical Information Network Clinical Trials Registry (UMIN‐CTR). The Trial registration number is ID: UMIN000052220. We report the trial results following the CONSORT 2010 Statement (Shulz et al., 2010).

### 3.2 Intervention

The intervention group received the CCOM program developed and previously pilot‐tested by the research team (Nagamatsu et al., 2025). The intervention was developed using the instructional design method (Gagne et al., 2004) to solve the difficulties experienced by nurses in the care coordination of PM patients. Instructional design is a systematic process of planning and delivering instruction based on an understanding of how learning occurs through internal cognitive processes (Gagne et al., 2004). Steps and processes of instructional design enable educators to determine the scope of the course contents, sequence of instructions, innovative presentation, and evaluation strategies (Obizoba, 2015). Instructional design identifies instructional problems, goals, learners' characteristics, content and context of instruction (Obizoba, 2015). Instructional design–driven programs improve nurses' knowledge, self‐efficacy, confidence, attitude, and clinical practice (Jia et al., 2025; Kim et al., 2020; Chu et al., 2019). In terms of PM care, which is a rare disease, nurses were inexperienced and unable to fully understand the care needs of PM patients, particularly those in the terminal stage. Nurses were unclear as to the care plans that they should implement as well as their roles within the fragmented healthcare system.

As for designing the instructions of the educational program, we adopted the Dick and Carey systems approach model (Dick et al., 2021). The CCOM program for nurses on the care coordination of PM patients consisted of two stages: (1) preparation and (2) face‐to‐face workshop. The preparation stage consisted of nine study videos featuring various aspects of PM (15 min each video) and a reading care guide as handout. The contents of the videos (2 h and 15 min) and face‐to‐face workshop (3 h and 30 min) are shown in Table 1. The face‐to‐face workshop included a lecture by a home care nurse regarding the care needs of PM patients and their families in the terminal stage at home using actual PM cases. The control group received no education.

**TABLE 1:** Contents of the educational program for nurses on the care coordination of pleural mesothelioma patients.

Preparation of study videos reading care guide as handout (2 h 15 min)	
Asbestos and mesothelioma	15 min
Surgery	15 min
Anti‐tumor therapy	15 min
Nursing care	15 min
Social service	15 min
Home nursing	15 min
Psychological support	15 min
Social benefits and peer support	15 min
Narrative of bereaved family of PM patient	15 min

Face‐to‐face workshop (3 hours 30 minutes)	
Introduction and ice‐breaking	30 min
Lecture by home care nurse:	30 min
Care needs of PM patients and their families in the terminal stage at home	
Group work: Care coordination plan for terminal case	90 min
Presentation	30 min
Activity introduction of advocacy group	20 min
Certificate award ceremony	10 min

### 3.3 Outcome measures

The three outcome measures [(1) Knowledge of disease and treatment of PM, (2) Attitude toward care coordination of PM patients, (3) Confidence in the care coordination of PM patients] were originally developed by the research team (Nagamatsu et al., 2025) based on the theoretical model of self‐efficacy by Bandura and Walters (1977) which was used as the framework for the present study to implement the educational program to improve nurses' care coordination skills. The development of these outcome measures was one of the steps in the instructional design method used to develop the CCOM Program. The instructional design model by Dick et al. (2021) consists of 10 steps, and Step 5 focuses on developing assessment instruments that evaluate learners' achievement of instructional objectives. Accordingly, nurses' confidence was measured corresponding to the 10 instructional goals identified in Step 1.

Items for the outcome measure: **(1) Knowledge of disease and treatment of PM** (*Knowledge*) were developed to assess the information required for nurses to achieve those goals. Items for the outcome measure. **(2) Attitude toward care coordination of PM patients** (*Attitude*) were constructed based on the difficulties reported by nurses providing care for PM patients (Nagamatsu et al., 2012) and the challenges identified during the feasibility study of the care guide (Nagamatsu & Sakyo, 2023). Items for the outcome measure. (3) **Confidence in the care coordination of PM patients** (*Confidence*) were based on the difficulties encountered by the nurses in the care coordination of PM patients.

To ensure content relevance, four nurses with clinical experience in PM reviewed the outcome measures. Based on their feedback, necessary modifications were made, and a pilot test was conducted to confirm the clarity and applicability of these evaluation tools or indicators (Nagamatsu et al., 2025). To assure content relevance, four nurses with clinical experience in PM reviewed the content and based on their comments, the necessary modifications were made and pilot‐tested (Nagamatsu et al., 2025).

The three outcome measures are detailed below:

**(1) Knowledge of disease and treatment of PM** (*Knowledge*): eight items (1 = right answer, 0 = wrong answer). The total score range is 0–8, and a higher score indicates higher knowledge. The knowledge scale consists of eight statements that measure knowledge in the following areas which are relevant to the care coordination of PM patients: (1) organ which develops mesothelioma, (2) pleurectomy‐decortication, (3) symptom to control after extra pleural pneumonectomy, (4) immune checkpoint inhibitors for PM, (5) occupations posing a risk of asbestos exposure, (6) benefit for PM caused by occupational exposure to asbestos, (7) asbestos health damage relief compensation system, and (8) side effects of immune checkpoint inhibitors in PM (Table 2). Participants select the answer(s) from the choices provided.

**TABLE 2:** Outcome measure: Knowledge of disease and treatment of PM.

1.	Choose the organ which does not develop mesothelioma
	a. Peritoneum
	b. Testicular serosa
	c. Pericardium
	d. Fascia
2.	Choose the correct description below about pleurectomy‐decortication
	a. Radiation therapy id often given after surgery
	b. It is a radical treatment for mesothelioma
	c. Less likely to cause atrial fibrillation than extrapleural pneumonectomy
3.	Choose the most important symptom that needs to be controlled after extrapleural pneumonectomy
	a. Hypoglycemia
	b. Constipation
	c. Loss of appetite
4.	Choose two immune checkpoint inhibitors approved for the treatment of PM
	a. Nivolumab
	b. Pembrolizumab
	c. Avelumab
	d. Ipilimumab
5.	Which occupations pose a risk of exposure to asbestos?
	a. Electrician
	b. Teacher
	c. Construction worker
	d. All of the above
6.	Choose the correct statement regarding PM caused by occupational exposure to asbestos
	a. Eligible for asbestos health damage relief compensation
	b. Workers' compensation can only be applied within 10 years after retirement
	c. Not eligible to apply for workers' compensation if one has been informed of the dangers of asbestos
7.	Which of the following statement is incorrect regarding the asbestos health damage relief compensation system?
	a. Applications can be made even after the patient's death
	b. You can receive almost the same compensation as workers' compensation
	c. Can apply for compensation at the public health center
	d. Patients with asbestos‐related lung cancer can also apply for compensation
8.	Choose the correct description about the side effects of immune checkpoint inhibitors in PM
	a. Frequent dermatitis
	b. To ensure effectiveness of the therapy, it is better to tolerate the effects as much as possible
	c. Steroids are contraindicated because the intestines and skin become hypersensitive

**(2) Attitude toward care coordination of PM patients** (*Attitude*): Original 10 items; 5‐point Likert scale (1 = strongly agree, 5 = never agree). The total score range is 10–50, and a higher score indicates a positive attitude. The original items were developed based on the attitudes of nurses who had difficulties in the care coordination of PM patients (Table 3). This scale showed Cronbach's α coefficients of 0.706 at pre‐test, 0.872 at post‐test, and 0.873 for the whole study.

**TABLE 3:** Outcome measure: Attitude toward care coordination of PM patients.

1	Pain in the terminal stage of PM cannot be controlled
2	It is difficult to ask PM patients and their families the intent for end‐of‐life care
3	I feel hesitant to contact a local medical facility and consult it about discharged PM patients
4	I am too busy to provide psychological care to PM patients and their family
5	I have no one to ask about PM care
6	It is a burden to suggest home care or palliative care to discharging PM patients
7	It is not the role of nurses to suggest about social benefits
8	It is not the role of nurses to introduce a patient support group
9	I cannot sympathize with PM patients and their family as regards their anger against asbestos
10	I do not know how to handle the victim feeling of PM patients

*Note*: 5‐point Likert scale (1 = strongly agree, 5 = never agree).

**(3) Confidence in the care coordination of PM patients** (*Confidence*): Original 10 items; 5‐point Likert scale (1 = not confident, 5 = confident). The total score range is 10–50, and a higher score indicates a higher confidence. Items are based on the difficulties encountered by the nurses in the care coordination of PM patients (Table 4). This scale showed Cronbach's α coefficients of 0.905 at pre‐test, 0.951 at post‐test, and 0.938 for the whole study.

**TABLE 4:** Outcome measure: Confidence in the care coordination of PM patients.

1	To interview newly diagnosed PM patients to check their understanding of the disease and treatment
2	To suggest social benefits to PM patients
3	To provide information about a patient support group
4	To provide care to manage the effects of surgery
5	To assess and manage the side effects of anti‐tumor therapy
6	To connect local care for discharging PM patients
7	To suggest home care or palliative care to discharging PM patients
8	To ask PM patients where they want to die
9	To suggest grief care to the family
10	To show empathy to the victim's feeling

*Note*: 5‐point Likert scale (1 = not confident, 5 = confident).

### 3.4 Evaluation of the CCOM program

Appropriateness of the program implementation method, appropriateness of the program duration, and usefulness in caring for PM patients were assessed using a 5‐point Likert scale (from agree to disagree). Additionally, participants were asked to provide open‐ended comments about the CCOM program.

### 3.5 Setting and participants

This study was conducted from November 2023 to December of 2023. The CCOM program for the intervention group was conducted three times in a university in Tokyo and once in a private hospital in Osaka from November 25 to December 16, 2023.

We carried out a purposive sampling of nurses who (i) have a nursing license and (ii) understand the Japanese language. After obtaining approval from our university review board, invitation forms were sent to the 24 hospitals that have an asbestos disease center.

**FIGURE 1:**
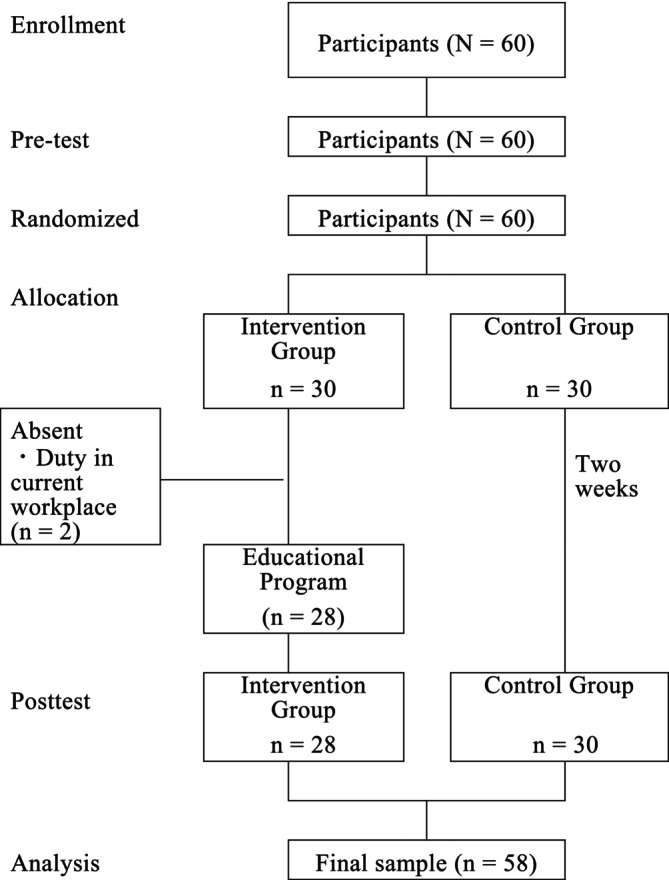
Flow diagram of randomization.

The flow diagram for randomization is shown in Figure 1. A total of 60 nurses agreed to participate in this study. Of these 60 nurses, two in the intervention group could not attend the face‐to‐face workshop because of work duty in their current workplace. Thus, a total of 58 nurses were evaluated.

### 3.6 Data collection

The participants initially completed a self‐administered questionnaire documenting their age, years of experience as a nurse, number of PM patients taken care of, and current work department during the time of their application to the educational program. Thereafter, the participants completed a self‐administered questionnaire of primary outcome at the pre‐test and post‐test. The intervention group answered at the post‐test form at the end of the education program, and the control group answered the post‐test 2 weeks after the pre‐test. Reminder mails were sent to the control group. Data were collected from the end of November 2023 to the middle of December 2023.

### 3.7 Sample size

The sample size for this study was calculated from the effects measured from a pilot study conducted by the research team (Nagamatsu et al., 2025). The pilot study was a single arm study (intervention group only) and 31 nurses answered questionnaire before, after, and 1 month later after the educational program. The sample size for this study was calculated with a significance level of .05, a power of 95%, and an effect size of 0.796 (attitude) which was the smallest among three measures. It was determined that 10 nurses or 5 per group would be required using G*Power software version 3.1 (Kang, 2021). To accommodate potential losses and because there were many nurses who wanted to participate in the CCOM program, the study enrolled a total of 60 nurses.

### 3.8 Allocation

Participants were randomly assigned in a 1:1 ratio to either the intervention or control group using random permuted block sizes of 4. The two groups were assigned so that there was no difference in the proportion of those with or without experience of care of PM. The randomization sequence was generated by the principal investigator (corresponding author) using the cloud service Mujinwari (https://autoassign.mujinwari.biz/).

### 3.9 Blinding

Participants were not blinded to the group allocation and intervention. All assessments were self‐reported and data analysis was blinded to the group allocation. Participants did not know which group they will be assigned to at the time of giving their consent for participation.

### 3.10 Statistical analysis

The distributions of the sociodemographic characteristics of the participants, such as age, years of clinical experience, and number of PM patients taken care of, were described and expressed as mean ± standard deviation. The participants' current workplaces (e.g., palliative care unit, pulmonary ward) were also described. A difference was considered significant when the corresponding *p* value was < .05. The distribution of all variables was checked for normality; nonparametric statistics were used when appropriate. The characteristics of the participants were compared between groups using the chi‐square test for categorical variables and the *t*‐test for continuous variables. Repeated measures multivariate analysis of variance was used to compare the total scores of the primary outcomes according to group, time, and group*time interaction. In the present study, “time” refers to the number of the test; that is, the pre‐test and the post‐test, not the actual time between the two tests. Data were analyzed using SPSS version 27.0.

### 3.11 Program evaluation analysis

Descriptive statistics were used to summarize the participants' quantitative ratings of the CCOM program. Inductive content analysis was conducted to analyze comments of open‐ended questions in the post‐test (Elo & Kyngäs, 2008). Three researchers who have experience of qualitative analysis independently read the comments and provided explanatory encoding for every sentence or paragraph related to the evaluation of the program. Then, each researcher generated categories and subcategories based on the similarity of each code. Thereafter, all researchers discussed the process for generating categories to enhance the trustworthiness of data analysis.

### 3.12 Ethical consideration

This study observed all standards for the protection of human subjects as set forth by the Declaration of Helsinki. The participants were informed that they could voluntarily stop their participation at any time without any questions or repercussions. The anonymity of their responses was also assured. Ethical approval for the study was obtained from the Research Ethics Committee of our university (approval number: 23‐A074).

## 4 RESULTS

### 4.1 Sociodemographic characteristics

We recruited 60 participants and randomly assigned them to two groups. All the participants completed the pre‐test and 58 completed the post‐test (Figure 1). The characteristics of the participants in the intervention and control groups are shown in Table 5. There were no significant differences in the means of the age and number of PM patients taken care of. However, the control group had a significantly longer nursing experience than the intervention group (*p* = .003). Nursing experience was adjusted as a covariate in two‐way repeated analysis of variance (ANOVA).

**TABLE 5:** Characteristics of participants in the intervention and control groups.

	Intervention group		Control group		*p* value
	*n* = 28		*n* = 30		
	Mean	SD	Mean	SD	
Age	39.1	10.8	37.9	9.9	*p* = .710
Nursing experience (year^a^)	8.9	6.9	12.9	0.2	*p* = .003
Number of MP patient taken care of	*n*	(%)	*n*	(%)	*p* = .844
0	17		17		
1–5	8		10		
6–10	1		2		
11<	2		1		
Working department					*p* = .963
Ward	11		8		
Home care/Community nursing	5		6		
Outpatient department	2		4		
Chemotherapy department	2		3		
Nursing administration	2		1		
ICU	1		2		
Palliative care	1		1		
Clinical trial	1		1		
OR	0		1		
Others (master student, faculty, midwife, consultant)	3		3		
Mean scores on measurements (pre‐test)					
Knowledge	3.7	1.6	3.5	1.4	*p* = .591
Attitude	32.6	4.2	31.4	5.6	*p* = .372
Confidence	23.4	7.9	20.8	8.2	*p* = .222

^a^ There was a significant difference in nursing experience years between the intervention and control groups.

### 4.2 Impact of the CCOM program on nurse’ knowledge, attitude, and confidence

A comparison of the mean scores of knowledge, attitude, and confidence between the intervention and control groups, as well as the results of the two‐way repeated‐measures ANOVA with covariate adjustment (nursing experience) are shown in Table 6.

**TABLE 6:** Confidence, attitude, and knowledge by group over time.

	Intervention group (*n* = 28)		Control group (*n* = 30)		Two‐way analysis of variance			
	Mean (SD)		Mean (SD)					
	Baseline	Post‐test	Baseline	Post‐test		Groups	Time	Group × Time
Knowledge^a^	3.7 (1.6)	7.3 (0.9)	3.5 (1.4)	3.8 (1.6)	*F*‐value	39.40	32.05	58.02
					*p*	<.001	<.001	<.001
					η^2^	0.42	0.37	0.51
Attitude^a^	32.6 (4.2)	40.0 (5.9)	31.4 (5.6)	31.9 (6.0)	F‐value	15.70	14.82	26.18
					*p*	<.001	<.001	<.001
					η^2^	0.22	0.21	0.32
Confidence^a^	23.4 (7.9)	40.3 (5.9)	20.8 (8.2)	24.3 (8.0)	*F*‐value	43.57	51.99	41.51
					*p*	<.001	<.001	<.001
					η^2^	0.44	0.49	0.43

^a^ Years of nursing experience were included as an adjusted covariate.

#### 4.2.1 Knowledge

The mean knowledge scores were not significantly different between the two groups at baseline; however, the intervention group showed significantly higher scores than the control group at the post‐test. The results of the two‐way repeated measures ANOVA test showed a significant large‐size effect of time on the participants' mean knowledge scores, indicating a significant change between the pre‐test and the post‐test (*p* < .001, *η* ^2^ = .37) across the two groups. The main effect of the educational program was significant (*p* < .001, *η* ^2^ = .42). There was a significant interaction between group and time (*p* < .001, *η* ^2^ = 0.51).

#### 4.2.2 Attitude

The mean attitude scores were not significantly different between the two groups at baseline; however, the intervention group showed significantly higher scores than the control group at the post‐test. The results of the two‐way repeated measures ANOVA test showed a significant size effect of time on the participants' mean confidence scores, indicating a significant change between the pre‐test and the post‐test (*p* < .001, *η* ^2^ = .21) across the two groups. The main effect of the educational program was significant (*p* < .001, *η* ^2^ = .22). There was a significant interaction between group and time (*p* < .001, *η* ^2^ = 0.32).

#### 4.2.3 Confidence

The mean attitude scores were not significantly different between the two groups at baseline; however, the intervention group showed significantly higher scores than the control group at the post‐test. The results of the two‐way repeated measures ANOVA test showed a significant large‐size effect of time on the participants' mean confidence scores, indicating a significant change between the pre‐test and post‐test (*p* < .001, *η* ^2^ = 0.49) across the two groups. The main effect of the educational program was significant (*p* < .001, *η* ^2^ = 0.44). There was significant interaction between group and time (*p* < .001, *η* ^2^ = 0.43).

### 4.3 Evaluation of CCOM program

#### 4.3.1 Quantitative evaluation of the CCOM program

The participants rated the CCOM program as appropriate in its implementation (96.3%) and duration (93.3%), and useful in caring for PM patients and their families (96.7%).

#### 4.3.2 Qualitative content analysis of evaluation comments

The results of the qualitative content analysis are shown in Table 7. The comments from the participants were classified into four categories: “Learning care needs of PM patients and families,” “Empathizing with PM patients and families,” “Efficiency of face‐to‐face workshop followed by preparation,” and “Gaining support to perform care coordination.” Participants gained understanding of the unique characteristics of PM, including its cause, rarity, fatality, specific treatments, and the psychological burden experienced by the patients and their families. Moreover, the CCOM program promoted the ability to empathize from the powerful narratives by family members and awareness of the universal risk of exposure to asbestos. Additionally, the participants reported that the CCOM program, which consisted of preparation and face‐to‐face workshop, minimized the time commitment. The group work also provided an opportunity to learn from other participants and to integrate knowledge to actual care. Furthermore, the participants gained support in conducting care coordination from other professionals and patient support groups, as well as from other nurses when facing care challenges.

**TABLE 7:** Categories and subcategories identified from the qualitative content analysis of evaluation comments.

Learning care needs of PM patients and families	Cause, rarity, and fatality of the disease
	Specificity of surgery and postoperative sequelae
	Understanding applicable pharmacotherapy
	Rapid disease progression at home
	Psychological burden of patients and families
Empathizing PM patients and families	Power of family's narrative
	Recognizing that universal risk of exposed to asbestos
Efficiency of face‐to‐face workshop followed by preparation	Time efficiency of preparation with handouts and videos
	Learning from other participants' experiences
	Integrating knowledge into care through group work
Gaining support to perform care coordination	Acquainting other professionals and patient support group
	Meeting nurses to consult when facing care challenges

## 5 DISCUSSION

### 5.1 Effectiveness of the CCOM program

For mesothelioma care, reports of care fragmentation remain limited. Warnock et al. (2019) reported that fragmented care increased anxiety in Great Britain. In Japan, PM patients who could not access care when their condition became critical at home were at a higher risk of not achieving a good death (Nagamatsu et al, 2022b) and their bereaved family members suffered complicated grief (Nagamatsu et al., 2022a). Few studies have addressed the barriers to the care coordination of mesothelioma in Japan and other countries. Previous studies have shown the following barriers to care coordination: fragmentation of the health service (Barrios et al., 2022; Klabunde et al., 2017); lack of experience of health care providers; lack of defined providers' roles (Hohmann et al., 2020); lack of a clear shared‐care plan (Hohmann et al., 2020); inadequate communication between a specialist and a primary caregiver (Walsh et al., 2010); reduced personnel (Barrios et al., 2022); lack of a comprehensive information sharing and communication system (Hohmann et al., 2020). The CCOM program was effective in improving the participants' knowledge, attitude, and confidence as the educational program was designed to show the roles of nurses using an actual case of a PM patient at the terminal stage, providing a care guide for PM patients to make up for inexperience, and providing opportunity to develop a support system with other nurses which empowers nurses to overcome various barriers.

The social learning theory by Bandura and Walters (1977) proposed that learning was possible not only by personal experience, but also by observing the actions and results of others. As nurses (1) have limited opportunities to experience providing care for patients with rare malignancies such as PM, and (2) even more rarely provide care for PM patients in the terminal stage at home, it is difficult to learn the care needs of PM patients in the terminal stage at home from their own experience. The CCOM program provided opportunities for the nurses to learn the care needs of PM patients and their families in the terminal stage from the perspectives of home care nurses. The evaluation comments indicated that the face‐to‐face workshop, preceded by preparation, enabled the participants to understand the care needs of PM patients at home, as well as the necessary care to meet those needs, and explore strategies for care coordination. Moreover, the collaborative efforts in care planning were significant, viewing them as a valuable platform for exchanging expertise and experiences related to care coordination. This process also facilitates the development of a support network, which is consistent with the principles outlined in Bandura's social learning Theory, thus emphasizing observational learning and modeling within a social context. All these components underlie the effectiveness of this educational program in improving the confidence of the nurses.

### 5.2 Limitations and future research

The effectiveness of the CCOM program was assessed only at two measurement points: pre‐test and post‐test. Future studies should include follow‐up assessments after a certain period to examine the sustained effects of the program.

In the current educational program, we could not obtain the contents of communication skills in the case of dying PM patients and their families because we needed to prioritize the understanding of their care needs in the terminal stage at home and the necessary care coordination to meet these care needs. However, communication skills are essential to the practice of care coordination (Khatri et al., 2023; Lisy et al., 2021; Walsh et al., 2010). Therefore, the development of educational programs that equally focus on advanced communication skills are essential.

The outcome measures examined in the pilot study demonstrated only internal consistency and content validity. Concurrent validity against an external criterion was not assessed. Future studies should evaluate criterion‐related validity and test–retest reliability and confirm the factor structure of these measures.

Finally, even if this educational program could improve the knowledge, attitudes, and confidence of the participating nurses, effective care coordination requires the ability to integrate all these three elements into care practice. The true effectiveness of an educational program must be evaluated based on the participants' future care coordination practices and the care satisfaction of PM patients and their families.

## 6 CONCLUSION

We conducted a randomized controlled trial to evaluate an educational program for nurses on the care coordination of PM patients (i.e., CCOM program). The results showed that the educational program for nurses significantly improved their knowledge, attitude, and confidence in the intervention group compared with the nurses in the control group without the educational program.

## AUTHOR CONTRIBUTIONS

**Yasuko Nagamatsu** had primary responsibility for conducting the study. **Nagamatsu** contributed to the conceptualization and study design, development and implementation of the educational program, data collection and analysis, drafting the initial version of the manuscript, and acquisition of funding. **Wakanako Ono and Keiko Hosokawa** contributed to the development of the educational program and conducted the qualitative analysis. **Shino Matsuura and Taiki Fukujin** contributed to the development and implementation of the educational program. **Yumi Sakyo** contributed to the qualitative analysis, data interpretation and critical revision of the manuscript. **Minako Ito** contributed to data collection, statistical analysis, and critical revision of the manuscript. **Masaya Ito and Satomi Nakajima** contributed to the development of the educational program and data interpretation. **Haruhiko Mitsunaga** contributed to the conceptualization and study design, as well as statistical analysi**s**. **Edward Barroga** contributed to English editing and critical revision of the manuscript. **Yoko Maehara, Nobukazu Fujimoto, and Kazunori Okabe** contributed to the development of the educational program and supervised the study. All authors read and approved the final manuscript.

## FUNDING INFORMATION

This work was supported by the Japan Society for the Promotion of Science Grants‐in‐Aid for Scientific Research (JSPS KAKENHI), grant number 23K21545.

## CONFLICT OF INTEREST STATEMENT

The authors declare that there is no conflict of interest.
